# The effects of timing of fine needle aspiration biopsies on gene expression profiles in breast cancers

**DOI:** 10.1186/1471-2407-8-277

**Published:** 2008-09-30

**Authors:** Vietty Wong, Dong-Yu Wang, Keisha Warren, Supriya Kulkarni, Scott Boerner, Susan Jane Done, Wey Liang Leong

**Affiliations:** 1 Department of Applied Molecular Oncology, Ontario Cancer Institute, Princess Margaret Hospital, University Health Network, Toronto, Canada; 2 Department of Radiology, Princess Margaret Hospital, University Health Network, Toronto, Canada; 3 Department of Pathology, Princess Margaret Hospital, University Health Network, Toronto, Canada; 4 Departments of Laboratory Medicine and Pathobiology Toronto, Canada; 5 Medical Biophysics, University of Toronto, Toronto, Canada; 6 Department of General Surgery, University Health Network, University of Toronto, Toronto, Canada; 7 Surgical Oncology, Princess Margaret Hospital, University Health Network, University of Toronto, Toronto, Canada

## Abstract

**Background:**

DNA microarray analysis has great potential to become an important clinical tool to individualize prognostication and treatment for breast cancer patients. However, with any emerging technology, there are many variables one must consider before bringing the technology to the bedside. There are already concerted efforts to standardize protocols and to improve reproducibility of DNA microarray. Our study examines one variable that is often overlooked, the timing of tissue acquisition, which may have a significant impact on the outcomes of DNA microarray analyses especially in studies that compare microarray data based on biospecimens taken *in vivo *and *ex vivo*.

**Methods:**

From 16 patients, we obtained paired fine needle aspiration biopsies (FNABs) of breast cancers taken before (PRE) and after (POST) their surgeries and compared the microarray data to determine the genes that were differentially expressed between the FNABs taken at the two time points. qRT-PCR was used to validate our findings. To examine effects of longer exposure to hypoxia on gene expression, we also compared the gene expression profiles of 10 breast cancers from clinical tissue bank.

**Results:**

Using hierarchical clustering analysis, 12 genes were found to be differentially expressed between the FNABs taken before and after surgical removal. Remarkably, most of the genes were linked to FOS in an early hypoxia pathway. The gene expression of FOS also increased with longer exposure to hypoxia.

**Conclusion:**

Our study demonstrated that the timing of fine needle aspiration biopsies can be a confounding factor in microarray data analyses in breast cancer. We have shown that FOS-related genes, which have been implicated in early hypoxia as well as the development of breast cancers, were differentially expressed before and after surgery. Therefore, it is important that future studies take timing of tissue acquisition into account.

## Background

DNA microarray analysis is an evolving high-throughput molecular technology that enables scientists to survey thousands of genes simultaneously. The resulting gene profiles (GPs) have been employed for the investigation of complex multi-factorial diseases such as breast cancer. In breast cancer, GPs have been shown to correlate with many clinically relevant clinico-pathological parameters, to prognosticate survival and predict treatment responses to specific chemotherapeutic regimens [1-9]. Despite its potential in clinical applications, one of the main hurdles to be overcome is the reproducibility of DNA microarray data due to several variables that may influence the results. These variables include different protocols used in tissue handling, RNA extractions and amplifications, microarray platforms and statistical analyses. Efforts are underway to standardize and validate the procedure. One such important initiative is the Microarray Quality Control (MAQC) project launched by the US Food and Drug Administration (FDA) [10]. It compared microarray data using identical RNA samples on three different DNA microarray platforms from independent laboratories. They demonstrated that in well designed studies under strict conditions, the microarray technology is highly reproducible. The FDA has recently approved several microarray platforms for clinical use.

In this study, we examined the hypothesis that timing of tissue acquisition, either taken pre- or post-operatively (*in vivo *or *ex vivo*, respectively), is one of the important variables that may influence DNA microarray results. Many studies have failed to report or control for this variable. It is particularly important when comparing studies designed to evaluate gene profiles of *in vivo *specimens such as those obtained in the neo-adjuvant setting, to more traditional studies that evaluate gene profiles in *ex vivo *specimens from surgery or tissue banks.

Fine needle aspiration biopsies (FNABs) have been used successfully to obtain cancer cells for DNA microarray studies [11-17]. As compared with core biopsy or surgically resected specimens, FNAB specimens appear to enrich for epithelial cells with comparable yield in RNA [11,12]. Additional advantages of using FNABs include the ability to obtain specimens for microarray analyses without significant compromise of standard histological assessments, and the ability to assess cytology of these FNAB specimens.

In this study, we compared GPs from FNABs taken pre-operatively and post-operatively in 16 patients to determine the effects of timing on the microarray data. To identify genes that may potentially confound with gene signatures in DNA microarray data.

## Methods

### Patients and fine needle aspirate biopsies (FNAB)

Sixteen patients with known palpable invasive breast cancer planned to have surgery for the primary tumors and nodal assessment, were recruited into the study at Princess Margaret Hospital (Toronto, On). This study was approved by our institutional research ethics committee, University Health Network Research Ethics Board, and all patients gave written informed consent. In each of the 16 patients, the first FNAB was taken before surgery (PRE) and the second FNAB was taken immediately after the surgical removal of the tumor in the operating room (POST). FNABs were obtained using a 25-gauge needle on a 10-cc syringe, with the needle passing the tumor 10 to 20 times. Two needle biopsies were utilized for each time point. An aliquot of the FNAB was placed into 15 ml of CytoLyt (Cytyc Corp; Marlborough, MA) for cytological analyses by the cytopathologist (SB). The remaining cells in the FNABs were placed into 500 μl of RNA extraction lysis buffer (Qiagen; Valencia, CA) and were immediately lysed by repeatedly passing the suspension through the FNAB needle and then quick frozen to -80°C and stored for later processing. Table 1 summarizes the clinico-pathological features of the 16 patients, FNAB cytology and RNA yields from each of the two time points.

**Table 1:** Tumor clinico-pathological features, cytology, and RNA yields of the patients.

Patient No.	Tumor Type	Tumor Size (cm)	Tumor Grade	ER*	PR*	HER2*	Positive Nodes	PRE Total RNA (μg)	PRE Tumor Cell (%)	POST Total RNA (μg)	POST Tumor Cell (%)
1	IDC	2.8/2.6	2 & 3	+	+	+	2–26	9.6	>99	15.4	>99
2	IDC	1.9	3	+	+	-	0/12	0.8	>99	1.4	>99
3	IDC	2	3	+	+	-	1–17	0.4	>99	9.8	>99
4	IDC	1.6	3	+	+	-	1–4	1.9	>99	1.4	>99
5	IDC	3	2	+	-	-	0/16	0.2	>99	1	**
6	IDC	15	3	+	+	-	29/32	0.5	>99	1.8	>99
7	IDC	4.5	3	-	-	+	0/11	7.6	>99	30.4	>99
8	IDC	1.6	2	+	+	-	0/16	1.4	>99	3.7	>99
9	IDC	1.7	3	-	-	-	1–18	2	>99	1.1	>99
10	IDC	2.2/1.0	3	-	-	+	2–17	7.5	>99	3.4	>99
11	IDC/lobular	6.5	2	+	+	-	8–15	1.5	>99	3.2	>99
12	IDC	2.5	3	-	-	-	8–25	1.3	**	12.4	>99
13	IDC	4	2	+	+	-	2–20	4.2	>99	8.5	>99
14	IDC	6.5	2	-	-	+	1–12	9.5	>99	3.2	>99
15	IDC	2.1	2	+	+	-	0/3	1.2	>99	9.6	50
16	IDC	3.4	2	+	+	-	0/3	1.9	>99	0.4	>99

ER, estrogen receptor; PR, progesterone receptor; HER2, Her2/neu receptor status; IDC, invasive ductal carcinoma; ILC, invasive lobular carcinoma.*ER, PR, and HER2 status as reported on standard surgical pathology reports were determined by immunohistochemistry, or by fluorescent in situ hybridization (FISH) for HER2 according to our institutional standard of practice. ** Cytology from FNABs of patient 5 POST and 12 PRE could not be determined due to low cellularity in the cytologic fractions.

### Tissue bank specimens

For comparison purposes, we obtained 10 fresh frozen random invasive breast cancer specimens from our institutional tissue bank (University Health Network Biobank). The tissue bank specimens were annotated with clinico-pathology information and taken from patients with breast cancers larger than 2 cm who had surgery at our institution.

### RNA extraction and amplification

Frozen FNAB lysates were thawed in a 37°C water bath for 15 min, and RNA was extracted using RNAeasy Micro kit (Qiagen). 15–20 mg of tissue bank specimens were placed into 700 μL of RNA extraction lysis buffer (Qiagen) and then homogenized using a PRO 200 rotor stator homogenizer (PRO Scientific Inc; Oxford, CT). The RNA was extracted using RNAeasy Lipid mini kit (Qiagen). Qualitative analyses of the RNA samples were determined using the Agilent 2100 Bioanalyzer RNA 6000 LabChip kit (Agilent Technologies; Palo Alto, CA). In many cases the RNA yields from the FNABs were less than 2 μg (Table 1), to standardize the RNA processing, we amplified all the RNA samples using the MessageAmp aRNA amplification kit, a T7 based linear amplification (Ambion; Austin, Tx) in one round. 100–1000 ng of total RNA was used as a starting amount for amplification according to the manufacturer's instructions.

### Microarray hybridization

The amplified RNA species from FNABs and an amplified Universal Human Reference RNA (Stratagene; La Jolla, CA) were directly labeled using cyanine 3 and cyanine 5 fluorescent dyes for microarray hybridization. Direct labeling of the sample on glass slide Microarrays was preformed according to the protocol given by the University Health Network Microarrays Centre (University Health Network, Toronto, On) [18]. Briefly, first-strand cDNA synthesis was performed with Superscript II (Invitrogen; Carlsbad, CA) in the presence of cyanine 3-dCTP or cyanine 5-dCTP (Amersham; Little Chalfont, UK) from 5 to 10 μg of RNA. The generated cDNA probes were then purified and denatured. The labeled targets were hybridized to UHN h19k cDNA microarrays (single spotted cDNA microarray chips comprising of 19,008 characterized and unknown human ESTs) manufactured at the University Health Network Microarray Centre. Scanning of the microarrays was achieved by using the Axon Scanner 4000A (Molecular Devices Corp.; Sunnyvale, CA) to obtain 16 bit TIFF image files.

### Microarray and statistical analysis

GenePix 6.0 (Molecular Devices Corp.) was used to analyze the TIFF image files of the h19k cDNA microarrays for quantitation. A total of 64 microarray image files of pre (PRE) and post-operative (POST) samples with dye-swap replicates from the 16 patients were analyzed. The raw data in GenePix report (GPR) format were directly transferred into Acuity 4.0 (Molecular Devices Corp.) for analysis. After Lowess slide normalization of the GPR files, any uninformative microarray data were flagged and filtered. The dye-swap signal ratios were inverted before the data of the replicate were combined. Probes with minimal changes in expression level (Log2 ratio >= 1 or <= -1 in not more than 2 tumors) were removed, resulting in a list of 4,056 probes (National Center for Biotechnology Information Gene Expression Omnibus depository [19], accession number GSE12072). Then t-Test and hierarchal clustering analyses were used to select differentially expressed genes. Subsequent statistical analyses were carried out using SPSS 13.0 (SPSS, INC., Chicago, IL). Pearson Correlation was used to measure the expression level similarities between the replicated microarrays as well as between the microarray and real-time quantitative RT-PCR (qRT-PCR) examinations. PathwayAssist 3.0 (Stratagene) was used to determine the gene product interactions, based on a reference database (Ariadne Inc. Rockville, MD), updated in October 2007. Downloaded annotation files of UHN h19k cDNA microarray were updated by using UniGene Human Build 211 (GPL7025).

### Real-time quantitative RT-PCR(qRT-PCR)

The expression levels of selected genes in the microarray data were validated by performing qRT-PCR on the amplified RNA samples that were used for the microarray hybridization. qRT-PCR was performed using Gene Amp 9700 sequence detector (Applied Biosystems, Foster City, CA). Pre-designed Taqman gene expression assays (Applied Biosystems) were used for each gene using primers away from the 3' end sequence of the transcript. For all qRT-PCR reactions, standard concentration of assays and Universal TaqManPCR Mastermix (Applied Biosystems) were used. The Sequence Detection Software (Applied Biosystems) was used to obtain the amplification plot to quantify gene expression values using the cycle threshold method. Human universal Reference RNA (Stratagene) was used as the calibrator sample, and the housekeeping gene GAPDH served for the standardization of the individual PCR reactions.

## Results

### Comparison of Pre and Post-operative microarray profiles

**Figure 1:**
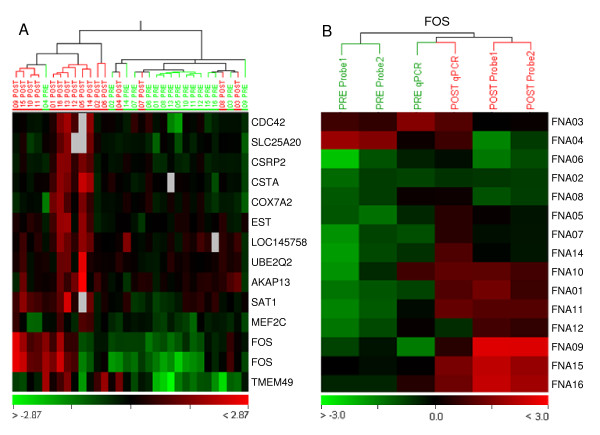
**Differential gene expression pattern of breast cancers between FNABs taken pre-operatively (PRE) and post-operatively (POST)**. (A) Hierarchical clustering pattern of expression of 14 cDNA probes between PRE and POST FNABs (p < 0.05, t-test). Rows represent cDNA probes and columns represent FNAB samples. "PRE" and "POST" correspond to pre and post-operative biopsies for the respective samples. The expression level is depicted according to the color scale (bottom) which represents the Log2 ratio changes of the samples relative to the human universal reference RNA (Stratagene). Grey squares indicate missing or filter-excluded data. (B) Validation of *FOS *gene expression in cDNA microarray using qRT-PCR. qRT-PCR data shown are averages of duplicate measurements. The columns correspond to FOS expression data in the microarray and qRT-PCR from PRE and POST specimens. The rows represent the corresponding specimens. Each cell in the matrix represents the expression level of Log2 ratio for microarray or Log ratio for qRT-PCR relative to the human universal reference RNA (Stratagene).

After data filtering (see Methods section), a list of 4,056 cDNA probes remained informative. We then preformed a t-test on the log2 ratio of each cDNA probe to identify genes discriminating PRE and POST samples from each patient by using a P-value < 0.05 detection level. Then a hierarchical clustering analysis was used to separate the relatively homogeneous clusters. As a result, 14 cDNA probes representing 12 unique genes and one expressed sequence tag of an unknown gene (Fig. 1A) were identified (see Additional file 1). Two cDNA probes corresponding to FOS were found to be most significant in differentiating PRE and POST specimens (P = 0.002 and 0.006).

### Biological relationships of differentially expressed genes from Pre and Post-operative microarray profiles

**Figure 2:**
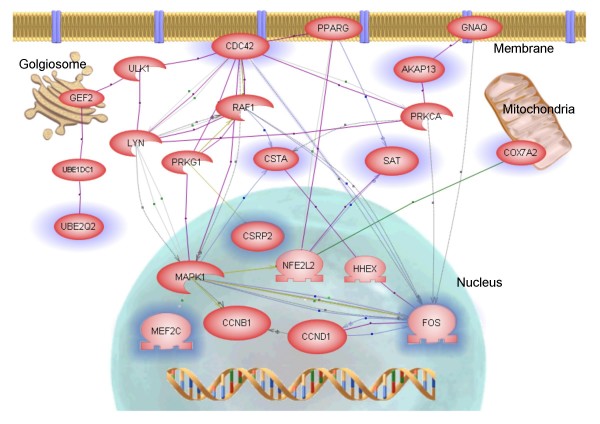
**Pathway linkage analysis of the differentially expressed genes between PRE and POST FNABs**. Nine genes (blue halo), out of 11 differentially expressed genes, including FOS were linked in the analysis (PathwayAssist 3.0) and formed a network. The layout of cellular location for the proteins is graphically presented.

The biological relationships of the 12 genes differentiating PRE and POST GPs were further investigated using PathwayAssist 3.0 software (Stratagene). We searched using the shortest paths between the genes to make up their biological interaction network. The software recognized 11 of the 12 genes. Nine out of the 11 genes formed a network and they were linked directly or indirectly to FOS (Fig. 2). qRT-PCR was used to validate the expression level of FOS in each of the FNAB specimens (Fig. 1B).

### Trends in FOS expression with prolonged ischemia

**Figure 3:**
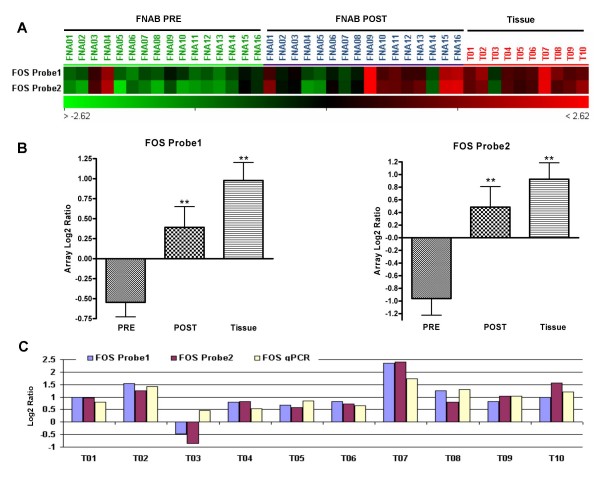
**FOS expression in PRE, POST and tissue specimens of breast cancers**. (A) The expression levels of two FOS probes in microarray data of PRE and POST FNAB from 16 cases of invasive breast cancers, and tissue specimens from 10 invasive breast cancers. (B) FOS expression changes among the different specimens for array probe 1 (PRE vs. POST, P = 0.002 and PRE vs. tissue, P = 0.000023) and array probe 2 (PRE vs. POST, P = 0.006 and PRE vs. tissue, P = 0.000071). (C) Comparison of FOS expression levels between microarrays and qRT-PCR data for tissue specimens of breast cancers. The correlation coefficients between FOS expression level detected by qRT-PCR and two array data for tissue specimens are 0.86 (P = 0.0015) and 0.79 (P = 0.0063).

FOS is known to be expressed in hypoxic conditions [20], and we hypothesized that what we observed in the higher expression of multiple members of the FOS network was related to the length of ischemic exposure prior to biopsy. To test this hypothesis, we compared the expression levels of FOS in 10 surgical specimens of invasive breast cancers that were left in an ischemic state for at least 30 minutes before freezing (Figure 3) to our FNAB specimens. The cDNA microarray analyses showed that FOS expression levels in the tissue samples were indeed higher than that in PRE and POST FNAB specimens (P = 0.000023, p = 0.000071) (Fig 3A and 3B) with a trend of higher FOS expression with longer ischemic time. Figure 3C showed the concordance between the microarray expression levels of the two FOS probes and the qRT-PCR data in tissue samples.

## Discussion

DNA microarray is an evolving technology that has promise in diagnosing and prognosticating clinical outcomes of breast cancers [1-9]. This has led to increased efforts to standardize this technology [10]. Implementing standards for microarray technology involves controlling all the potential confounding variables systematically, from sample collection to microarray data management, to ensure reliability of test results. Our study examined one such variable that has not been addressed in previous studies, which is the timing of tissue acquisition for DNA microarray analyses.

In our study, we elected to use FNAB for tissue acquisition. When compared to core biopsy, FNAB appeared to be less invasive for the participating patients, and the cell specimens collected were mostly malignant cells (99% table 1). The latter finding is consistent with a study by Symmans *et al*. [11] in which they reported enrichment of epithelial cells in FNAB as compared with core biopsy specimens. The loose cells collected appeared to improve RNA extraction from small biopsy specimens as it facilitates cell lysis without the need for tissue homogenization and with minimal contamination of other cellular components. Although the RNA yield is variable (0.2 to 30.4 ug, table 1), overall, sufficient quantity and quality of RNA can be obtained for the purpose of molecular profiling when amplified. Several studies have assessed the fidelity of T7 based linear RNA amplifications and have shown that linear amplification with as little as 100 ng-1000 ng is highly representative [21-23]. Due to variable RNA yields from FNABs, the qRT-PCR component of this study was performed on amplified RNA which may introduce 3' bias as a result of the amplification process. However, we did not observe significant 3' bias as we were able to correlate our microarray data even though the qRT-PCR primers were selected from sequences away from the 3' end of the transcripts. A number of gene expression profiling studies have also been able to validate their data using amplified RNA in qRT-PCR [16,24-29].

Gene profiles generated from the FNABs taken before and after surgery in general were reproducible except for the 12 genes related to FOS. Since FOS has previously been associated with ischemic conditions [20,30,31], we hypothesized that this observation was due to the duration of exposure to hypoxia. We then tested the hypothesis by demonstrating a much higher FOS expression level, using DNA microarray and qRT-PCR, in fresh frozen tissue specimens that were exposed to a longer ischemic time (figure 3A, B and 3C). Our pre-operative tumor FNABs were performed on tumors that were still *in vivo *and the specimens were immediately placed in lysis buffer and snap frozen after the biopsy. The post-operative FNABs were obtained similarly but on *ex vivo *tumors after surgical excision in the operating room. In the case of tissue bank specimens, fresh surgical specimens were delivered to the pathology laboratory before freezing and therefore consistently experienced hypoxia much longer than the FNAB specimens. Müller et al [20] reported that hypoxia can induce FOS expression as early as 15 minutes exposure to hypoxia but plateaus at about 30 minutes. This was consistent with our findings which demonstrated a higher level of FOS expression with longer ischemic time (Fig. 3).

Hypoxia can induce two classes of hypoxia-related genes: immediate early genes (induced within minutes) and delayed response genes (arising slowly over hours) [32]. FOS is an example of an immediate early gene, it complexes with JUN to form the activator protein-1 transcription factor complex (AP-1) which regulates gene expression in response to hypoxia, and it has been known to be associated with regulation of cell cycle activities such as cell proliferation, differentiation, transformation, and apoptotic cell death [30]. In classic hypoxia pathways activated through Hypoxia-Inducible Factor-1α (HIF1α), the response to hypoxia is delayed [32], these genes include vascular endothelial growth factor (VEGF) [33], erythrpoietin (EPO) [34], neuronal nitric oxide synthase (NOS) [35], endothelin 1 (EDN1) [36], lactate dehydrogenase A (LDHA) [37] glycolytic enzymes and glucose transporters such as glycolytic enzymes aldolase A(ALDA) and phosphoglycerate kinase 1 (PGK1) [38], Many of these hypoxia-related pathways have been implicated in adverse cancer biology and may be linked to chronic hypoxia and tumor necrosis [39].

From the 4,056 informative genes in our microarray data, we were not able to detect changes in gene expressions related to other hypoxic pathways before and after the surgery. Out of the twelve differentially expressed genes, only FOS had potential interactions with HIF pathway. FOS interacts with HIF1α via AP-1 complex [32,40]. It is possible that in surgical specimens, the expressions of immediate early genes induced by hypoxia, including FOS, were elevated minutes after removal of the tumors. However, expressions of delayed response genes, such as those activated by HIF1α, were not significantly altered because they required a longer time to elicit any significant change. This would be consistent with our findings of elevated gene expressions in FOS-related hypoxia pathway but not in other hypoxic pathways mediated through HIF1α. However, given the limitations of our study, we are not able to rule out all the changes in other hypoxic pathways. After data filtering (see Methods section), some of the critical genes in these pathways, including HIF1α and VEGF, were not represented in our microarray data. Further study is needed to draw concrete conclusion on the other hypoxic pathways from our observations.

In addition to the effects of hypoxia, there was a possibility that the physiological responses to general anesthesia or the stress of the surgery could have caused the observed changes in tumor gene expression, if this was the case, the mechanism is currently unknown and it would appear to be mediated through FOS-related pathway.

Interestingly, FOS has been associated with breast cancer in a number of studies [41-44]. FOS is involved in the expression of genes associated with malignant progression such as metalloproteases that degrade the extracellular matrix that may facilitate invasion and metastasis [45-47]. Most previous microarray studies utilized banked tissue [1-4] and hypoxia-related genes may influence the result of the clustering analyses by affecting the ranking of differentially expressed genes. For example, Sotiriou *et al*. [7] reported that FOS was one of the genes that separated basal 1 and 2 subtypes of breast cancers, although it is difficult to know if the group could draw a similar conclusion if they used *in vivo *specimens in their study instead. In contrast to the study by Sotiriou *et al*., Perou *et al*. [4] examined gene expression profiling on breast cancer tissue (unpublished data cited in [4]), they excluded a cluster of genes including FOS simply because the authors believed that these genes were induced by prolonged handling of samples following surgical resection. It is quite possible that early hypoxic genes such as FOS may affect the ranking of some genes in previously published gene signatures. Hence, timing of tissue acquisition must be considered when interpreting microarray data.

## Conclusion

Our study demonstrated that expression of early hypoxic genes can be influenced by the timing of tissue acquisition. Notably FOS, which is one of the most differentially expressed genes between the two time points was implicated in both hypoxia [20,31,32,40] and other breast cancer gene signatures [4,7,41]. Our study suggested that the timing of sample collection may confound microarray data analysis and therefore must be taken into consideration when designing future studies.

## Abbreviations

FNAB: Fine Needle Aspiration Biopsy; PRE: Pre-operative; POST: Post-operative; GP: gene profiles; MAQC: Microarray Quality Control; FDA: US Food and Drug Administration; GPR; GenePix Report; ER: Estrogen Receptor; PR: Progesterone Receptor; Her2: Her2/neu receptor; IDC: Invasive Ductal Carcinoma; ILC: Invasive Lobular Carcinoma; RT: reverse-transcriptase; PCR: polymerase chain reaction; FOS and JUN are proto-oncogenes, AP-1: activator protein-1 transcription factor complex; HIF1α: hypoxia-inducible factor-1α; VEGF: vascular endothelial growth factor; EPO: erythrpoietin; NOS: neuronal nitric oxide synthase; EDN1: endothelin 1; LDHA: lactate dehydrogenase A; ALDA: glycolytic enzymes aldolase A; PGK1: phosphoglycerate kinase 1.

## Competing interests

The authors declare that they have no competing interests.

## Authors' contributions

VW participated in the design, experimentation, data collection, analysis, interpretation, research and wrote this manuscript. DYW participated in the study design, data analysis, and preparation of the table and figures for this manuscript. The interpretation of the cytology samples was performed by SB. SK performed the FNABs in the radiology suite. WLL conceived the study design and was involved in the data collection, interpretation and manuscript preparation as well as supervision of the overall project. KW provided technical expertise with the experiments. Critical revision of the manuscript was performed by VW, DYW, WLL, SJD. All authors read and approved the final manuscript.

## Pre-publication history

The pre-publication history for this paper can be accessed here:



## Supplementary Material

Additional file 1 DNA Microarray data of genes differentiating PRE and POST FNAB samples. Genes differentially expressed between PRE and POST FNABs in DNA microarray analysis. Click here for file
